# Pedometers and Aerobic Capacity: Evaluating an Elementary After-School Running Program

**DOI:** 10.1155/2014/370759

**Published:** 2014-03-02

**Authors:** Elizabeth Wanless, Lawrence W. Judge, Shannon T. Dieringer, David Bellar, James Johnson, Sheli Plummer

**Affiliations:** ^1^School of Physical Education, Sport, and Exercise Science, HP 360P, Ball State University, Muncie, IN 47304, USA; ^2^School of Kinesiology, University of Louisiana at Lafayette, Lafayette, LA 70504, USA

## Abstract

Childhood obesity affects 1 of every 6 youth in the United States. One contributing factor to this statistic is a lack of physical activity (PA). Demands related to accountability which are placed on educators to demonstrate academic achievement often result in resistance to allocating time during the school day for PA. One possible solution is to consider utilizing time after school to integrate PA programs. The purpose of this study was to assess the impact of a 12-week after-school pedometer-focused PA program on aerobic capacity and to examine the relationship between step count and aerobic capacity in elementary school aged children. A group of elementary students (*n* = 24; 9.5 ± 0.9 years) participated in a 12-week pedometer-focused PA program that included pretraining and posttraining fitness testing via the 20-meter version of the PACER test. Paired sample *t*-tests revealed significant differences between the pretest (*M* = 21.0 laps, *SD* = 9.9) and posttest (*M* = 25.2 laps, *SD* = 12.2) scores (*t* = 4.04, *P* ≤ 0.001). A Pearson correlation revealed no significant relationship between individual step count and the difference between PACER pre- and posttest (*r* = 0.318, *P* = 0.130). The program improved aerobic capacity, but an increase in pedometer-calculated step count was not a predictor.

## 1. Introduction

Obesity-related risk factors and diseases are becoming increasingly prevalent in pediatric populations. Over the past thirty years, the obesity rate in the United States has escalated, creating an epidemic (15% to 30% in adults, 5% to 18% in adolescents 12–19 years, and 6% to 19% in children 6–11 years) [1]. Obesity has been linked to numerous medical complications (e.g., hypertension, stroke, certain types of cancer, and coronary heart disease), as well as lower cognitive performance and reductions in brain structural integrity [2]. Furthermore, the impact of obesity on the development of type 2 diabetes is so profound that the onset of this disease is now befalling American youth. The approach to address these health concerns may include alternative strategies which focus more on prevention rather than on treatment. Given that the effects of obesity and inactivity begin during childhood and multiply as children reach adulthood, prevention may be the most effective strategy to address these growing concerns. Having identified a concern regarding inactivity and obesity, the next step may be to identify and implement effective intervention strategies. One such strategy is the development of programs through partnerships with schools. As such, this strategy may provide the framework needed to implement prevention strategies through increased physical activity (PA) and education.

Research suggests that regular participation in PA plays an important role in sustaining good health and has been a topic of investigation for several decades [3]. The Center for Disease Control (CDC) via the US Department of Health and Human Services recently outlined PA guidelines for American youth. These guidelines recommend that pediatric populations (ages 6–17) should engage in 60 minutes or more of moderate to vigorous PA daily with vigorous activity occurring a minimum of 3 days per week [3–5]. The CDC also suggests that the PA be aerobic and focus on muscle and bone-strengthening activities [3–5]. The American College of Sports Medicine [5] developed the guidelines further by suggesting four areas of focus: cardiorespiratory exercise, resistance exercise, flexibility exercise, and neuromotor exercise.

The need for enhancing appropriate PA levels in order to reverse increasing trends of obesity and the prevalence of other health-related diseases associated with physical inactivity is at an all-time high [6–8]. Previous research has indicated that most American youth do not meet the recommended CDC guidelines [9, 10]. Additionally, researchers have reported that pediatric populations who engage in the recommended “dose” of PA are at lower risk of cardiovascular diseases and diabetes and have increased muscle and bone strength, cognitive and brain functioning, and psychosocial and mental health [2, 4, 11–14]. Finally, pediatric populations who participate in PA are more likely to engage in habitual PA and sport [15]. Additionally, research studies have linked the amount of PA engaged during pediatric years with PA levels during adolescence and adulthood [16].

In addition to the health benefits, academic achievement has also been linked to PA levels. Research indicates that daily PA improves concentration and academic achievement and can enhance test scores in math, reading, and writing [17, 18]. For these reasons, researchers have urged school administrators to implement long-term school-based interventions in order to prevent and manage childhood obesity [19]. The implementation of PA programming will ideally benefit learning and achievement for students. Several studies have stated that providing increased time for PA (e.g., physical education and after-school PA programs) can lead to better concentration, reduced disruptive behaviors, and higher test scores in reading, math, and writing [20]. It has also been proven that when students are involved in a PA program there is an “improved rate of academic learning per unit of class time” [21] and that increased time spent in physical education does not negatively affect student scores.

Although the research suggests that the recommended “dose” of PA increases health and academic performance, only 8% of elementary schools, 6.4% of middle schools, and 5.8% of high schools provide daily physical education to all of their students [22]. Additionally, 20% of all elementary schools in the USA have eliminated recess in favor of increased classroom time under pressure to improve student achievement [20], making viable solutions during the school day seemingly limited. In an effort to identify effective strategies to optimize student achievement, many school administrators are seeking to provide alternative programs, which engage students, provide social/emotional outlets, and increase PA as a means to improve the overall student performance. One alternative strategy that school administrators are beginning to utilize is after-school programming.

Limited research has been conducted on the effects of after-school programs on increasing PA levels of the pediatric participants involved [23]. With a dearth of data from school-based interventions, the overall results may initially seem less promising than anticipated; and the efficacy is yet still undefined [24]. In a review of the literature conducted by Pate and O'Neill [23], twelve after-school programs were evaluated. The authors concluded that the findings were mixed in regard to increasing PA levels of each of the programs studied. However, each intervention method, conversely, resulted in the formation of some type of healthful lifestyle habit. Additionally, Carrel et al. [25] investigated the effects of a 9-month after-school PA program on body composition and cardiovascular fitness of elementary school children. Both body composition and cardiovascular fitness improved significantly for obese and nonobese pediatric participants. An analysis by Gonzales-Suarez et al. [19] showed that long-running programs were more effective in preventing childhood obesity if they combined PA and classroom learning; in fact, the longer the program, the more effective it proved to be. Although there is a need for further investigation and understanding of after-school programs, the preliminary evidence supports promoting after-school programming to increase PA levels and increase aerobic capacity of pediatric populations.

In order to further investigate the benefits of after-school PA programs, it is important to use a validated measurement system. The use of pedometers to chart PA also continues to grow in popularity. Pedometers have gained widespread acceptance among PA researchers over the past decade [26]. A literature review conducted by Bravata et al. [27] evaluated 26 studies with a total of 2767 adult pedometer-using participants. Participants increased steps by an average of 2,491 steps in comparison with a control that did not utilize pedometers. As a result, participants increased PA by an average of 26.9%, significantly decreased their body mass index by 0.38, and decreased blood pressure. Given these results, which demonstrate the effectiveness of pedometers coupled with the notion that pedometers can be inexpensive, there is an indication that pedometers may also be an effective and accurate tool for measuring PA in pediatric populations [28–30]. The purpose of this study was to assess the impact of a pedometer-focused 12-week after-school PA program on aerobic capacity as well as the relationship between step count and aerobic capacity in elementary school aged children.

## 2. Materials and Methods

### 2.1. Participants

A group of elementary students (*n* = 24), 9.5 ± 0.9 years (range 8 to 12)—the ratio between males and females was equally distributed—participated in and completed a pedometer-focused 12-week after-school PA program called *Step up for Health* (SUH). The SUH staff conducted outreach and registration activities with the aim of maximizing students' and parents'/guardians' knowledge of the program and increasing the convenience of the registration process. Prior to the beginning of testing, all procedures were approved by a local university's International Review Board (IRB). Once approved, parental consent forms were sent home with all potential participants (students' grades 3–6). Written and oral explanations (e.g., child assent) regarding procedures and potential risks to the participants were provided to parents from whom parental consent had been obtained. After a two-week period, the procedure was repeated to recruit more participants for the program. After obtaining parental permission and written or oral assent from each participant, baseline measurements were taken. A total of 34 students enrolled in the program but 10 dropped out for various reasons and did not complete the final assessment.

### 2.2. Procedures for the Step Up for Health After-School Running Program

This 12-week after-school running program was designed for elementary students in grades 3 through 6. The overall two-day-a-week program design was based upon a series of progressive walking/jogging workouts as well as PA centered games and activities. The program made use of the local school facilities to hold the biweekly, one-hour sessions. The types of activities and the format of each session were held consistent across the entire program. A typical session began with a meeting with all of the participants to explain what the schedule was for the day. Then, pedometers were passed out and the participants engaged in a warm-up activity (e.g., Kangaroo Tag), followed by group stretching. After the warm-up the participants engaged in a group discussion involving a “running tip” (e.g., controlled breathing) and then broke into preestablished teams and completed the prescribed running workout (e.g., 10-minute interval run). After the workout the participants completed a group activity (e.g., scavenger hunt) and a cool-down and recorded their pedometer steps. In addition, home workouts were provided to encourage participants to engage in PA at home and to promote exercise as a family activity.

Each participant was assigned a pedometer to wear in order to track his or her steps taken during each session of the program. A daily step goal was provided, and each participant was instructed to make daily pedometer step goals. The pedometer step counts were then calculated and converted to miles, allowing the participants to have a better understanding of the accomplishments. The supervisor of the program provided the participants with rules and guidelines on using their pedometer to ensure that the pedometer was being used appropriately. Additionally, personal journals were assigned to each participant in order to record step totals and document his/her thoughts and reflect on the activity session.

A licensed physical education teacher underwent a one-week training, conducted by an experienced university faculty in physical education, prior to the start of the program. The licensed physical education teacher with the support of university faculty supervised all sessions.

### 2.3. Procedures for Fitness Testing

The Cooper Institute created Fitnessgram in 1982 in order to provide physical education teachers with a convenient way to evaluate fitness and report fitness levels to students and parents. In 2012, the President's Council on Fitness, Sports and Nutrition (PCFSN) adopted the Fitnessgram as the new school-based PA and health promotion program. The assessments were created to evaluate the five areas of health-related fitness: cardiovascular fitness (aerobic capacity), muscular strength, muscular endurance, flexibility, and body composition [31].

One of the assessments, the progressive aerobic cardiovascular endurance run (PACER), also known as the multistage fitness test, was used to assess cardiovascular fitness. The PACER test has been shown to be a valid and reliable assessment related to a participant's VO2 max or aerobic capacity. The participants must jog/run 20 meters each time a signal sounds using the Fitnessgram Testing Administration CD. With the passing of each minute, the running time is reduced by one half second, encouraging the participants to increase their running speed and pace themselves with the length of time allowed [31]. Each participant can fail to reach the designated line, before the beep tempo sounds, once before being eliminated. The highest lap attained before failing to keep pace with the beep tempo is recorded as the score. This assessment was chosen as a measure for pre- and posttesting. Participants were familiarized with the test prior to the pretest trial.

During this test, each participant's score was recorded as the total number of laps. This assessment was conducted during week one and week twelve of the program to track improvements across the program. Prior to the beginning of the program, the licensed physical education teacher, who acted as the supervisor to the program, underwent a one-hour training to ensure that she was familiar with administering the test. The participants were briefed on best practices on calculating and recording PACER results. Each participant was paired with a peer during the test, and the participants were divided into smaller groups of 6–8 participants to ensure appropriate counting. Scoring of the test was performed with the assistance of undergraduate students enrolled in a physical education course of study. The student received training on the PACER test prior to being recruited to assist with the project.

### 2.4. Data Analysis

Data was first analyzed for normality via Shapiro-Wilk procedures and was not found to be significantly deviant from being normally distributed (*P* > 0.05). Subsequently, paired samples *t*-test was employed in order to assess differences in pre- and postworkout data. A Pearson bivariate correlation was utilized to determine the relationship between individual step count and the difference between PACER pre- and posttest data. Significance was set a priori to alpha <0.05 for all analyses, and all statistical analyses were performed using SPSS version 20.0.

## 3. Results

**Figure 1:**
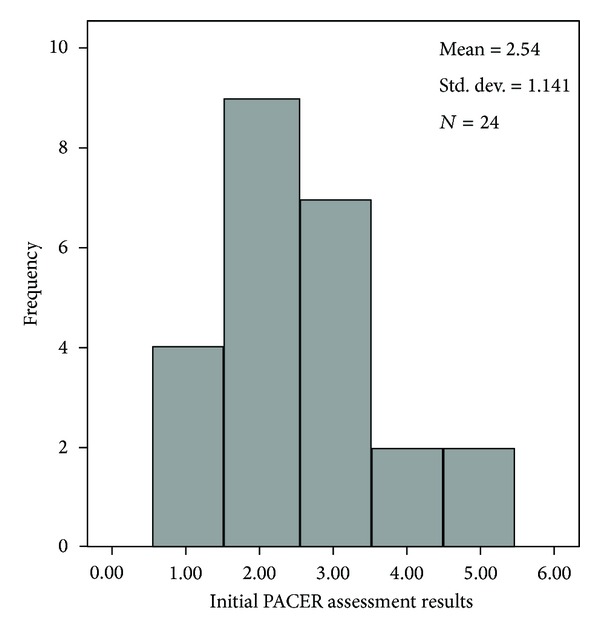
Histogram of initial 20-meter PACER assessment. Frequencies are groups by 1 (1–10 laps), 2 (11–20 laps), 3 (21–30 laps), 4 (31–40 laps), and 5 (greater than 40 laps).

**Figure 2:**
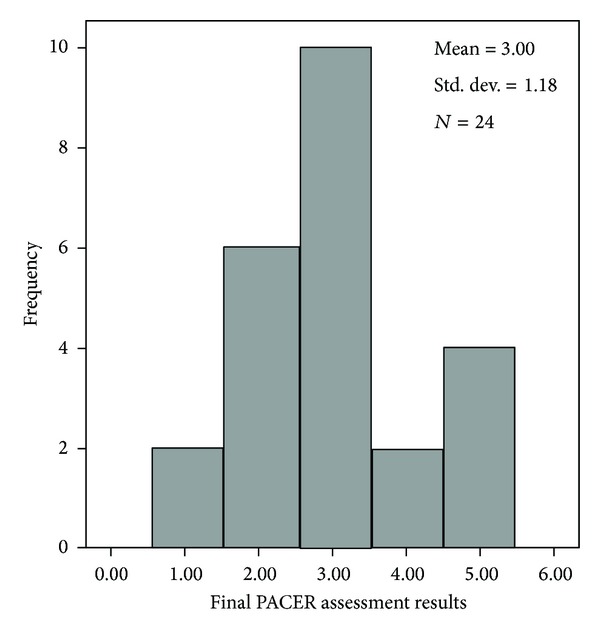
Histogram of final 20-meter PACER assessment. Frequencies are groups by 1 (1–10 laps), 2 (11–20 laps), 3 (21–30 laps), 4 (31–40 laps), and 5 (greater than 40 laps).

As a group, the elementary student participants totalled 1,388,155 steps for the project; individual steps ranged from 27,419 to 62,158 steps for the duration of the program (*M* = 47,867, *SD* = 9332). A paired sample *t*-test revealed significant differences between the PACER pretest (*M* = 21.0 laps, *SD* = 9.9) (Figure 1) and posttest (*M* = 25.2 laps, *SD* = 12.2) scores (*t* = 4.04, *P* ≤ 0.001) (Figure 2). These results indicate that there was improvement in aerobic capacity as a result of participating in the 12-week program. However, Pearson correlation revealed no significant relationship between the difference in individual step count and the difference between PACER pre- and posttest (*r* = 0.318, *P* = 0.130).

## 4. Discussion

This study evaluated the impact of a pedometer-focused 12-week after-school PA program on aerobic capacity as well as the relationship between step count and aerobic capacity in elementary school aged children. The following discussion addresses three topics: program effectiveness in terms of aerobic capacity, incorporating pedometers, and after-school programming as a solution to physical education decline while the obesity epidemic is on the rise.

### 4.1. Program Effectiveness in terms of Aerobic Capacity

Limited research has been conducted on the effects of after-school PA programs.Pate and O'Neill [23] reviewed twelve after-school programs and found mixed results with respect to the relationship between increased PA and improved aerobic capacity. The current study evaluated the effects of an after-school running program on pretest and posttest PACER scores, a widely accepted measure of aerobic capacity. The study results indicated that PACER scores significantly increased from program start to finish. Although this finding could not be compared with a control group of students, it does support previous conclusions from Pate and O'Neil [23], which found varied effects of after-school PA programs. Additionally, a study by Carrel et al. [25] indicated that after-school initiatives can improve cardiovascular fitness. In a related study by Judge et al. [32], fitness assessments were conducted via the PACER test of 76 elementary students who participated in an eight-week after-school training program. Paired sample *t*-tests revealed significant differences between the pretest (*M* = 11.9 laps, *SD* = 7.3) and posttest (*M* = 21.3 laps, *SD* = 11.5) on the PACER test.

### 4.2. Incorporating Pedometers

The results of this study demonstrate the positive impact of the after-school PA program on the aerobic capacity of the participants; however, step count did not predict improvement in aerobic capacity. This may be largely due to the variation in starting levels of fitness of the participants and the data collection of the pedometer being limited to only a volume of training assessment. Pedometers lack the ability to track intensity of movement which is critical to determining zones (light, moderate, and vigorous) of training. The results revealing that pedometer step count and aerobic capacity were not linked may seem contradictory to the overwhelming support for the use of pedometers as a tool to increase PA in adults [27]. However, Bravata et al. [27] showed several health benefits for adults as a result of pedometer use by way of increasing PA not directly related to increase in aerobic capacity. Further studies are needed to assess the value of pedometers as a tool to encourage increased PA and improved aerobic capacity in both adults and children.

Bravata et al. [27] acknowledged goal setting as one of the key determinants in participant PA increase and health benefit acquisition. Participant goals for steps per day, according to existing pediatric guidelines, are 15,000 steps/day for boys and 12,000 steps/day for girls [33]. These are reasonable targets for children with respect to improving health outcomes [33]. Perhaps one of the reasons why aerobic capacity did not increase as a result of pedometer-use was the neglect of program leaders to include goals for participants. A benchmark is needed for future running programs that include pedometers. In order to obtain that data, additional research is needed to more fully understand the impact of goal setting on increased PA levels for pediatric participants. Research concerning different levels of steps taken in order to comprehend how this type of goal setting may affect PA levels and aerobic capacity for pediatric participants would aid in setting a benchmark for future running programs incorporating pedometers.

Because pedometers have gained widespread acceptance, their accuracy in step count has also been a source of research study. Schneider et al. [34] tested accuracy among three brands in different conditions of walking among adult users. The authors reported a large variation in accuracy by brand. This may have limited the predictive value of the step count scores in the present investigation.

### 4.3. After-School Programming as a Solution

Schools have access to nearly all of a nation's youngsters. Developing strong partnerships with our schools may provide the framework needed to focus on the prevention through increased activity and education. Physical inactivity in American youth is an ongoing concern. It has been suggested that school-based programs be promoted to increase the opportunities for pediatric populations to engage in the recommended daily “dose” of PA. Although initiatives have been implemented and preestablished structures (e.g., physical education) provide an opportunity for pediatric populations to be active during the school day, students are still not meeting the recommendations. It seems that, with academic rigor being emphasized in schools, there is little time for PA. An attractive and fairly unexplored method to promote increased daily PA is structured after-school programming.

Although this study helped to confirm results of previous studies which indicate that after-school running programs have the potential to increase aerobic capacity and PA, after-school programs geared for twice a week in this study and three times a week for others fall very short of the CDC guidelines which recommend 60 minutes of exercise a day for all students attending the school [3]. However, in order for an afterschool running program to meet recommended CDC guidelines and produce the desired benefits, the program must be offered daily and students must be strongly encouraged to participate. This suggestion may be a further drain on school resources and, for that reason, may not be plausible.

The after-school programming to meet CDC requirements may not provide a total solution to address obesity and meet the needs of students, but certainly it would play an important role in meeting those needs. However, the culture of inactivity is more complicated than a simple solution of increased PA after-school can solve. Addressing the underlying behavioral and psychological mechanisms leading to the rising obesity trajectories is also an important step to solving the problem [32]. Further research is warranted in order to understand the effectiveness of an after-school PA/running program on the overall fitness of the participants.

## 5. Conclusions

The initial success of the SUH after-school running program as a service-learning project provides a working model for implementation of similar projects in the future. Although this project was focused upon implementation in an elementary school setting, application of these concepts by other academic units with similar goals of positively impacting their community through PA is recommended. According to the findings in this project, students' fitness levels can be improved through a program such as SUH that utilized pedometers as motivation to succeed. In this current study, the aerobic capacity of the students was improved even though pedometer step count was not a predictor. Future studies may wish to consider expanding to include all K-12 students and examine all aspects of fitness to better understand and promote healthy lifestyles in pediatric populations.
